# Innovative radiation oncology Together – Precise, Personalized, Human: Vision 2030 for radiotherapy & radiation oncology in Germany

**DOI:** 10.1007/s00066-021-01843-9

**Published:** 2021-09-13

**Authors:** David Krug, Markus Hecht, Nadja Ebert, Matthias Mäurer, Daniel F. Fleischmann, Emmanouil Fokas, Christoph Straube, Nils Henrik Nicolay, Juliane Hörner-Rieber, Daniela Schmitt, Cläre von Neubeck, Constantinos Zamboglou, Elena Sperk, David Kaul, Julia Hess, Stefanie Corradini, Clemens Seidel, Cihan Gani, Christian Baues, Benjamin Frey, Oliver Blanck, Tobias Gauer, Maximilian Niyazi

**Affiliations:** 1 Department of Radiation Oncology, University Hospital Schleswig-Holstein, Kiel, Germany; 2 Department of Radiation Oncology, Universitätsklinikum Erlangen, Friedrich-Alexander-Universität Erlangen-Nürnberg, Erlangen, Germany; 3 Department of Radiotherapy and Radiation Oncology, Faculty of Medicine and University Hospital Carl Gustav Carus, Technische Universität, Dresden, Germany; 4 Department of Radiotherapy and Radiation Oncology, University Hospital, Friedrich-Schiller-University, Jena, Germany; 5 Department of Radiation Oncology, University Hospital, LMU Munich, Munich, Germany; 6 Department of Radiotherapy and Oncology, University Hospital, Goethe University, Frankfurt, Germany; 7 Department of Radiation Oncology, Klinikum rechts der Isar, Technical University of Munich, School of Medicine, Munich, Germany; 8 RadioLog, Hof, Germany; 9 Department of Radiation Oncology, Medical Center—University of Freiburg, Faculty of Medicine, University of Freiburg, Freiburg, Germany; 10 Department of Radiation Oncology, Heidelberg University Hospital, Heidelberg, Germany; 11 Department of Radiation Oncology, University Medical Center Göttingen, Göttingen, Germany; 12 Department of Particle Therapy, University Hospital Essen, University of Duisburg-Essen, Essen, Germany; 13 Department of Radiation Oncology, University Medical Center Mannheim, Medical Faculty Mannheim, Heidelberg University, Mannheim, Germany; 14 Department of Radiation Oncology, Charité—Universitätsmedizin Berlin, Corporate Member of Freie Universität Berlin, Humboldt-Universität zu Berlin, Berlin, Germany; 15 Research Unit Radiation Cytogenetics, Helmholtz Zentrum München, German Research Center for Environmental Health GmbH, Neuherberg, Germany; 16 Klinik für Radioonkologie und Strahlentherapie, Universitätsklinikum Leipzig, Leipzig, Germany; 17 Department of Radiation Oncology, Eberhard Karls Universität Tübingen, Tübingen, Germany; 18 Department of Radiation Oncology and Cyberknife Center, University Hospital of Cologne, Cologne, Germany; 19 Department of Radiotherapy and Oncology, University Medical Center Hamburg-Eppendorf, Hamburg, Germany

**Keywords:** Vision development, Radiation therapy, Image guidance, Precision oncology, Career development

## Abstract

**Purpose:**

Scientific and clinical achievements in radiation, medical, and surgical oncology are changing the landscape of interdisciplinary oncology. The German Society for Radiation Oncology (DEGRO) working group of young clinicians and scientists (yDEGRO) and the DEGRO representation of associate and full professors (AKRO) are aware of the essential role of radiation oncology in multidisciplinary treatment approaches. Together, yDEGRO and AKRO endorsed developing a German radiotherapy & radiation oncology vision 2030 to address future challenges in patient care, research, and education. The vision 2030 aims to identify priorities and goals for the next decade in the field of radiation oncology.

**Methods:**

The vision development comprised three phases. During the first phase, areas of interest, objectives, and the process of vision development were defined jointly by the yDEGRO, AKRO, and the DEGRO board. In the second phase, a one-day strategy retreat was held to develop AKRO and yDEGRO representatives’ final vision from medicine, biology, and physics. The third phase was dedicated to vision interpretation and program development by yDEGRO representatives.

**Results:**

The strategy retreat’s development process resulted in conception of the final vision “Innovative radiation oncology Together – Precise, Personalized, Human.” The first term “Innovative radiation oncology” comprises the promotion of preclinical research and clinical trials and highlights the development of a national committee for strategic development in radiation oncology research. The term “together” underpins collaborations within radiation oncology departments as well as with other partners in the clinical and scientific setting. “Precise” mainly covers technological precision in radiotherapy as well as targeted oncologic therapeutics. “Personalized” emphasizes biology-directed individualization of radiation treatment. Finally, “Human” underlines the patient-centered approach and points towards the need for individual longer-term career curricula for clinicians and researchers in the field.

**Conclusion:**

The vision 2030 balances the ambition of physical, technological, and biological innovation as well as a comprehensive, patient-centered, and collaborative approach towards radiotherapy & radiation oncology in Germany.

**Supplementary Information:**

The online version of this article (10.1007/s00066-021-01843-9) contains supplementary material, which is available to authorized users.

During recent years, physical, technological, and clinical developments have led to improvements in radiation therapy precision, minimally invasive surgery, molecular targeted drugs, and immunotherapy. Radiation therapy plays an essential role in various multidisciplinary cancer treatment concepts in nearly all tumor entities in both curative and palliative intent. In order to further guide future development and strengthen the role of radiation oncology, the German Society for Radiation Oncology (DEGRO) working group of young clinicians and scientists (yDEGRO) and the DEGRO representation of associate and full professors (AKRO) initiated the development of a vision for the future of radiotherapy & radiation oncology in Germany.

The process of vision development, its chronology, methodology, and the one-day strategy retreat held by the yDEGRO and AKRO is presented in Fig. 1. The final vision, “Innovative radiation oncology Together – Precise, Personalized, Human” (original German phrase *Innovative Radioonkologie im Team – Präzise, Personalisiert, Menschlich*) and the respective interpretation and program are summarized in Tables 1 and 2. Background material regarding the vision development process as well as supporting statements by the Working Group Radiation Oncology (ARO) of the German Cancer Society, the German Society of Medical Physics (DGMP), the German Society of Radiobiology Research (DeGBS), and the Professional Association of German Radiation Oncologists (BVDST) are given in the online supplement.

**Fig. 1:**
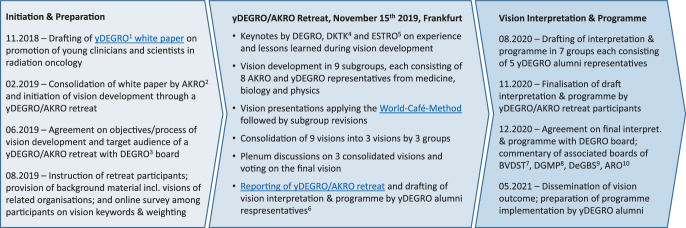
Development process of German radiotherapy & radiation oncology vision “Innovative radiation oncology Together – Precise, Personalized, Human.” ^1^German Society for Radiation Oncology (*DEGRO*) working group of young clinicians and scientists and ^6^its alumni representatives as a link between young scientists and executives/leaders in radiotherapy and radiation oncology in Germany. ^2^Representation of associate and full professors of the DEGRO. ^3^German Society for Radiation Oncology. ^4^German Consortium for Translational Cancer Research. ^5^European Society for Radiotherapy and Oncology. ^7^Professional Association of German Radiation Oncologists. ^8^German Society of Medical Physics. ^9^German Society of Radiobiology Research. ^10^Working Group Radiation Oncology of German Cancer Society. This figure contains hyperlinks highlighted in blue, which are accessible through the corresponding figure version in the supplementary material. (https://link.springer.com/article/10.1007%2Fs00066-021-01843-9#Sec1)

**Table 1:** Interpretation and program of the German radiotherapy & radiation oncology vision 2030 “Innovative radiation oncology Together – Precise, Personalized, Human”

	Innovative radiation oncology	Together	Precise	Personalized	Human
*Interpretation*	– Focus on preclinical research and clinical, prospective, randomized, multimodal, practice-oriented trials – Development of new, individualized treatment concepts – Identification of new indications	– Internal team building involving all radiation oncology professions (radiation oncologists, medical physicists, radiation biologists, radiation therapists, nursing, and administrative staff) – Interdisciplinary team building with other oncological and medical disciplines as well as associated non-medical professionals – Multi-layered interaction with professional societies and other relevant regulatory, political, and funding organizations – Sustainable promotion of young scientists and future scientific leaders	– Combination of radiation therapy with molecular targeted tumor therapeutics – Image-guided adaptive radiation therapy – Standardized and evidence-based technological quality requirements	– Individualized treatment in view of biological, medical and personal characteristics and needs of patients – Implementation of molecular, clinical, and imaging-based biomarkers – Individualized treatment through processing of dosimetric and image data from clinical trials and routine clinical practice	– Focus on the patient and her/his family – Define therapy goals taking into account the patients’ ability to make decisions – Participation and promotion of “personalities” within the respective professions – Balancing of work and family life
*Program*	– Forming of a national committee for strategic development in radiation oncology research – Promotion of clinical evidence by: 1. Developing a national trial registry. 2. Developing a national radiation oncology health service registry. 3. Expansion of prospective controlled trials and clinical registry trials – Expansion and coordination of third-party funded research – Prompt implementation of clinical trial outcomes through rapid adaptation of guidelines (cf. NCCN) – Development of national quality standards on implementing new technologies – Expanding professorships of basic research and of translational research	– Promotion of open communication in a respectful, creative working environment. Strengthening and empowering team members for intense exchange both within and between the radiation oncology professions. – Interdisciplinary, cooperative, and self-confident representation of radiation oncology in relation to medical and non-medical collaborative partners and political or regulatory bodies – Resident training rotation in cooperating disciplines (e.g., diagnostic radiology, internal medicine, palliative care) for sustainable interdisciplinary team building – Development of personalized and contractually secured programs for the sustainable qualification of future scientific leaders – Promotion of defined career paths/positions with focus on patient care, research, and teaching – Expansion of clinician scientist and mentoring programs; monitoring for relevant political aspects; clinical training program for radiation biologists – Promotion of inspiring medical, physical, biological teaching as a central mechanism to fascinate students for a career in radiation oncology	– Extension of combination therapy towards targeted tumor therapeutics – Further development of imaging techniques for image-guided radiation therapy – Establishment of national programs for clinical assessment of high-end technologies – Promotion of industrial technology development based on clinical needs and evidence – Establishment of national concepts and collaborations for machine learning and artificial intelligence – Standardized procedures in data acquisition of clinical data and study registries	– Focus on comprehensive approach to oncology – Acquisition of the patient’s treatment history, the biology of the disease as well as individualized treatment goals into national database – Development of scores for patient classification based on large database analysis – Promoting integration of high-quality, translational research in the design of clinical trials (bench to bedside and back) – Testing and evaluation of new combination therapies	– Patient-centered communication as part of the resident training in radiation oncology – Systematic and continuous recording of treatment relevant side effects and symptom relief by the patient-reported outcomes (PROs) approach to quantify quality of life – Promotion of supporting programs (exercise, diet, etc.) – Comprehensive psycho-oncological support for patients – Development of dedicated work models to ensure work–life balance – Consideration of parenthood as an integral part of the academic career

**Table 2:** Interpretation und Programmatik der Zukunftsvision 2030 „Innovative Radioonkologie im Team – Präzise, Personalisiert, Menschlich“ der Strahlentherapie & Radioonkologie in Deutschland

	Innovative Radioonkologie	Im Team	Präzise	Personalisiert	Menschlich
*Interpretation*	– Fokussierung auf präklinische Forschung sowie, prospektive, randomisierte, multimodale und praxisrelevante klinische Studien – Entwicklung neuer individualisierter Therapiekonzepte – Identifikation neuer Indikationen	– Fachinterne Teambildung der strahlentherapeutischen Berufsgruppen (Strahlentherapeuten, Medizinphysiker, Strahlenbiologen, MTRA, MFA und Pflegepersonal) – Fachübergreifende Teambildung mit anderen onkologischen, medizinischen und nicht-med. Fächern – Vielschichtige Interaktion mit Fachgesellschaften/Fachverbänden und anderen politischen, regulatorischen Instanzen und Förderinstitutionen – Nachhaltige Förderung des wissenschaftlichen Nachwuchses und wissenschaftlicher Führungskräfte	– Kombination mit molekular zielgerichteten Tumortherapeutika – Bildgeführte adaptive Strahlentherapie – Standardisierte und evidenzbasierte technologische Qualitätsanforderungen	– Therapieindividualisierung hinsichtlich biologischer, medizinischer und persönlicher Merkmale und Bedürfnisse der Patienten – Implementierung von molekularen, klinischen und bildgebenden Biomarkern – Individualisierung der Behandlung durch dosimetrische und bildgebende Datensätze aus klinischen Studien und Routineverfahren	– Patienten und Angehörige in den Mittelpunkt stellen – Therapieziele festlegen unter Beachtung der Entscheidungsfähigkeit der Patienten – Partizipation und Förderung von „Persönlichkeiten“ innerhalb der Berufsgruppen – Vereinbarkeit von Beruf und Familie
*Programmatik*	– Etablierung eines nationales Gremiums zur Strategieentwicklung für radioonkologische Forschung – Steigerung der klinischen Evidenz durch: 1. Etablierung eines nationalen Studienverzeichnisses. 2. Etablierung eines nationalen, strahlentherapeutischen Versorgungsregisters. 3. Ausbau von prospektiven kontrollierten Studien und klinischen Registerstudien – Ausdehnung und fachspezifische Koordinierung der Drittmittelforschung – Zeitnahe Implementierung klinischer Studienergebnisse durch rasche Adaption der Fachempfehlungen (vgl. NCCN) – Entwicklung nationaler Qualitätsstandards zur Implementierung neuer Technologien – Ausbau von Lehrstühlen für Grundlagenforschung und Lehrstühlen mit translationaler Ausrichtung	– Förderung einer offenen Kommunikation in einer respektvollen, kreativen Arbeitsumgebung. Stärkung und Befähigung von Teammitgliedern zum intensiven Austausch sowohl innerhalb, als auch zwischen den strahlentherapeutischen Berufsgruppen – Fachübergreifende, kooperative und selbstbewusste Repräsentation der Radioonkologie gegenüber medizinischen und nicht-medizinischen Kooperationspartnern sowie politischen oder regulatorischen Instanzen – Rotation in kooperierende Disziplinen (z. B. diagnostische Radiologie, Innere Medizin, Palliativmedizin) für eine nachhaltige fachübergreifende Teambildung – Entwicklung von personalisierten und vertraglich gesicherten Maßnahmen zur nachhaltigen Ausbildung von wissenschaftlichen Führungskräften – Förderung von definierten Karrierewegen/Planstellen, die eine Schwerpunktbildung bzw. eine Kombination aus Patientenversorgung, Forschung und Lehre vorsehen – Ausbau von Clinician-Scientist- und Mentoring-Programmen; Monitoring für fachpolitische Fragen; klinisches Hospitationsprogramm für Strahlenbiologen – Förderung einer begeisternden medizinischen, physikalischen, biologischen Lehre als zentralen Mechanismus zur Gewinnung von Nachwuchs für die Strahlentherapie	– Weiterentwicklung der Kombinationstherapie in Richtung zielgerichteter Tumortherapeutika – Weiterentwicklung von bildgebenden Verfahren für die bildgeführte Strahlentherapie – Etablierung nationaler Programme zur Analyse und Bewertung des klinischen Nutzens moderner Technologien – Förderung einer industriellen Technologieentwicklung mit Bezug auf klinischen Bedarf und Evidenz – Etablierung nationaler Konzepte und Kooperationen zum Einsatz von maschinellem Lernen und künstlicher Intelligenz – Standardisierte Erfassung von klinischen Daten und Studiendaten	– Fokus auf ganzheitlichen Aspekt der Onkologie – Erfassung der Biologie der Erkrankung, der Anamnese sowie der persönlichen Behandlungsziele der Patienten in einer bundesweiten Datenbank – Entwicklung von Scores zur Patientenklassifizierung auf Grundlage großer Datenbanken – Förderung der Integration hochwertiger translationaler Forschung in das Design klinischer Studien (bench to bedside and back) – Erprobung und Prüfung neuer Kombinationstherapien	– Patientenzentrierte Gesprächsführung als Teil der radioonkologischen Facharztausbildung – Systematische und kontinuierliche Erfassung therapierelevanter Nebenwirkungen und von Symptomlinderung im Sinne von Patient Reported Outcomes (PROs) zur Quantifizierung der Lebensqualität – Förderung von therapie-begleitenden Programmen zur Bewegung, Ernährung etc. – Umfassende psychoonkologische Betreuung der Patienten – Entwicklung von spezifischen Arbeitszeitmodellen zur Vereinbarkeit von Beruf und Familie – Berücksichtigung von Familienzeiten als integrativem Bestandteil einer akademischen Laufbahn

The interpretation of the term “Innovative radiation oncology” highlights the role of designing and initiating clinical trials and translational research, the development of individualized treatment concepts, and the identification of new indications for radiotherapy. The critical implementation steps are formation of a national strategy committee for radiation oncology research and the expansion and coordination of third-party funded research.

The term “Together” reflects the aspect of team building within radiation oncology professions, i.e., radiation oncologists, medical physicists, radiation biologists, radiation therapists, and nursing staff. Additionally, “Together” emphasizes interdisciplinary team building among radiation oncology professionals, medical oncologists, physicians from other medical disciplines, and associated non-medical professionals, e.g., data scientists. The term also implies and emphasizes the promotion of young scientists and future scientific leaders. The central work program includes strengthening the multidisciplinary radiation oncology team and promoting closer collaborations both within and beyond radiation oncology departments, i.e., with cooperation partners. This considers a resident training rotation in cooperating disciplines, expanding clinician scientist programs, and establishing mentoring programs to inspire and encourage young researchers for a scientific career in translational research. Furthermore, the role of promoting inspiring medical, physical, and biological teaching is highlighted as a central mechanism to fascinate students for a career in radiation oncology.

The term “Precise” covers both the technical precision of image-guided adaptive radiation therapy and treatment with molecular targeted drugs within the context of multimodal oncologic treatments. The key work program involves expansion of imaging techniques and standardization of data acquisition and its use for approaches to artificial intelligence.

The term “Personalized” focuses on individualized treatment strategies that take into account biological, medical, and personal information and patients’ needs. It also promotes the development of molecular, clinical, and imaging-based biomarkers for diagnosis, response evaluation, and prognosis. The key work program includes establishing a database for multicentric collection of clinical and biological data from routine practice and clinical trials. It also addresses the integration of high-quality translational research into the design of clinical trials (from bench to bedside and back). Data analysis and classification are based on methods of medical informatics.

The term “Human” underlines the need for a continuous patient-centered approach in radiation oncology. Innovation in radiation oncology serves to improve overall patient outcome and quality of life. Patients are empowered to make conscious, informed decisions about their treatment. Furthermore, this term emphasizes the promotion of “personalities” within the respective professions and acknowledges the need to promote flexible solutions and individual career paths to balance work and family life and open up long-term academic career pathways that cater to individual needs and strengths. The key work agenda contains the establishment of a resident training program for patient-centered communication and the facilitation of comprehensive programs for psycho-oncologic support, diet counselling, and physical exercise for cancer patients. Patient-reported outcomes (PROs) should be regularly measured in clinical trials and clinical routine.

In conclusion, the vision 2030 for radiotherapy & radiation oncology in Germany reconciles the ambition of physical, technological, and biological innovation as well as a comprehensive, patient-centered, and cooperative approach in oncology.

## Supplementary Information

Supplement 1: Documents related to the DEGRO/AKRO strategy retreat.

Supplement 2: Supporting statements from ARO, BVDST, DeGBS and DGMP.

Supplement 3: Development process of German radiotherapy & radiation oncology vision including embedded hyperlinks.

