# Correction to: Innovative radiation oncology Together—Precise, Personalized, Human

**DOI:** 10.1007/s00066-021-01869-z

**Published:** 2021-11-03

**Authors:** David Krug, Markus Hecht, Nadja Ebert, Matthias Mäurer, Daniel F. Fleischmann, Emmanouil Fokas, Christoph Straube, Nils Henrik Nicolay, Juliane Hörner-Rieber, Daniela Schmitt, Cläre von Neubeck, Constantinos Zamboglou, Elena Sperk, David Kaul, Julia Hess, Stefanie Corradini, Clemens Seidel, Cihan Gani, Christian Baues, Benjamin Frey, Oliver Blanck, Tobias Gauer, Maximilian Niyazi

**Affiliations:** 1 Department of Radiation Oncology, University Hospital Schleswig-Holstein, Kiel, Germany; 2 Department of Radiation Oncology, Universitätsklinikum Erlangen, Friedrich-Alexander-Universität Erlangen-Nürnberg, Erlangen, Germany; 3 Department of Radiotherapy and Radiation Oncology, Faculty of Medicine and University Hospital Carl Gustav Carus, Technische Universität, Dresden, Germany; 4 Department of Radiotherapy and Radiation Oncology, University Hospital, Friedrich-Schiller-University, Jena, Germany; 5 Department of Radiation Oncology, University Hospital, LMU Munich, Munich, Germany; 6 Department of Radiotherapy and Oncology, University Hospital, Goethe University, Frankfurt, Germany; 7 Department of Radiation Oncology, Klinikum rechts der Isar, Technical University of Munich, School of Medicine, Munich, Germany; 8 RadioLog, Hof, Germany; 9 Department of Radiation Oncology, Medical Center—University of Freiburg, Faculty of Medicine, University of Freiburg, Freiburg, Germany; 10 Department of Radiation Oncology, Heidelberg University Hospital, Heidelberg, Germany; 11 Department of Radiation Oncology, University Medical Center Göttingen, Göttingen, Germany; 12 Department of Particle Therapy, University Hospital Essen, University of Duisburg-Essen, Essen, Germany; 13 Department of Radiation Oncology, University Medical Center Mannheim, Medical Faculty Mannheim, Heidelberg University, Mannheim, Germany; 14 Department of Radiation Oncology, Charité—Universitätsmedizin Berlin, Corporate Member of Freie Universität Berlin, Humboldt-Universität zu Berlin, Berlin, Germany; 15 Research Unit Radiation Cytogenetics, Helmholtz Zentrum München, German Research Center for Environmental Health GmbH, Neuherberg, Germany; 16 Klinik für Radioonkologie und Strahlentherapie, Universitätsklinikum Leipzig, Leipzig, Germany; 17 Department of Radiation Oncology, Eberhard Karls Universität Tübingen, Tübingen, Germany; 18 Department of Radiation Oncology and Cyberknife Center, University Hospital of Cologne, Cologne, Germany; 19 Department of Radiotherapy and Oncology, University Medical Center Hamburg-Eppendorf, Hamburg, Germany

**Correction to:**

**Strahlenther Onkol 2021**

10.1007/s00066-021-01843-9

The original version of this article unfortunately contained mistakes. The name of the author Hess was corrected. Fig. 1 contained orthographic errors. Additionally, to ensure that the hyperlinks embedded in Fig. 1 can be accessed, Fig. 1 was added as Supplement 3. A reference to Supplement 3 has been added in the description of Fig. 1 in the article.

The original article has been corrected.

