# Supplementary material for: Innovative radiation oncology Together – Precise, Personalized, Human: Vision 2030 for radiotherapy & radiation oncology in Germany
Source: Strahlenther Onkol. 2021 Sep 13;197(12):1043–8. doi: 10.1007/s00066-021-01843-9 (PMC8604860; doi:10.1007/s00066-021-01843-9)
Supplement: Supplementary file 1 — Supplement 1: Documents related to the DEGRO/AKRO strategy retreat. [file 66_2021_1843_MOESM1_ESM.pdf]

1. Gemeinsames Treffen der DEGRO AGs Akademische Radioonkologie und Junge DEGRO

15. November 2019 im VIP-Bereich des Stadions des FSV Frankfurt

Übergeordnetes Ziel gemeinsamer AG Treffen
Dialog der Generationen in der akademischen Radioonkologie

Ziel des ersten Treffens
Entwicklung einer Zukunftsvision* der Strahlentherapie & Radioonkologie in Deutschland

Agenda

11:00 – 11:15

(I) Eröffnung durch unsere Moderatorin Ilse Neuenhofen, Unternehmensberatung being to inspire

(II) Grußworte von Stephan Siegler, Stadtverordnetenvorsteher der Stadt Frankfurt am Main

(III) Grußworte von Stephanie Combs, AKRO-Sprecherin und Matthias Mäurer, jDEGRO-Sprecher

11:15 – 12:00 Impulsreferate

(I) Dt. Gesellschaft für Radioonkologie – Wo kommen wir her? (Rita Engenhart-Cabillic / Claus Belka)

(II) Visionsentwicklung des Deutschen Konsortiums für Translationale Krebsforschung (Stefan Joos)

(III) Way to ESTRO Vision 2030 and Lessons Learned (Vincenzo Valentini)

(IV) Online-Umfrage zu Schlüsselwörter für die Zukunftsvision der dt. Radioonkologie (David Krug)

13:00 – 14:45 Gruppenarbeit mit Moderation durch AKRO Mitglieder mit den Zielen:

(I) Diskussion der Schlüsselwörter, Formulierung und Begründung der Zukunftsvision

(II) Interpretation der Zukunftsvision für die Teilbereiche Fachgesellschaft, Forschung, Weiterbildung und Patientenversorgung

15:00 – 17:00 Präsentation der Gruppenarbeit, Ergebnissicherung und Konsensfindung im Plenum

* Die Zukunftsvision soll u.a. ein Idealbild unseres Fachs zeichnen und der Frage nachgehen, was wir in den kommenden 10 Jahren erreichen wollen. Aus der Zukunftsvision sollen langfristige Ziele und Schwerpunkte für unsere Patientenversorgung, Weiterbildung, Forschung und Fachgesellschaft abgeleitet werden können.

Umfrage zu Schlüsselwörtern & Gewichtung zur Visionsentwicklung auf jDEGRO/AKRO-Treffen

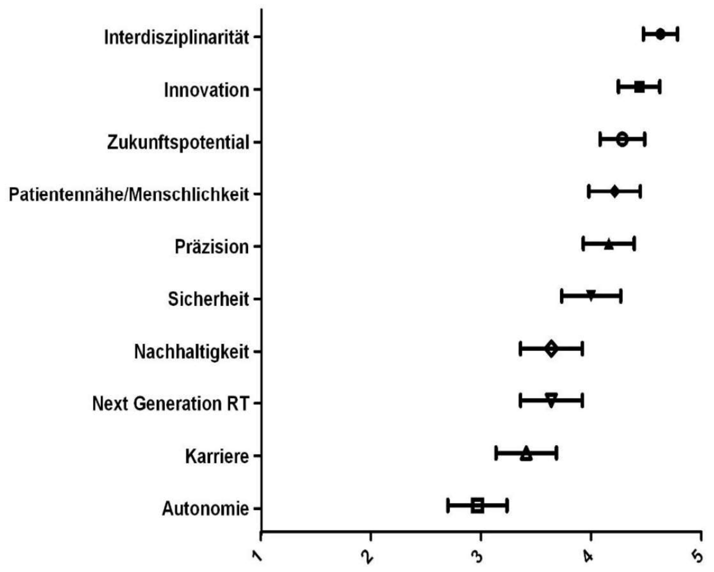

Übergeordnetes

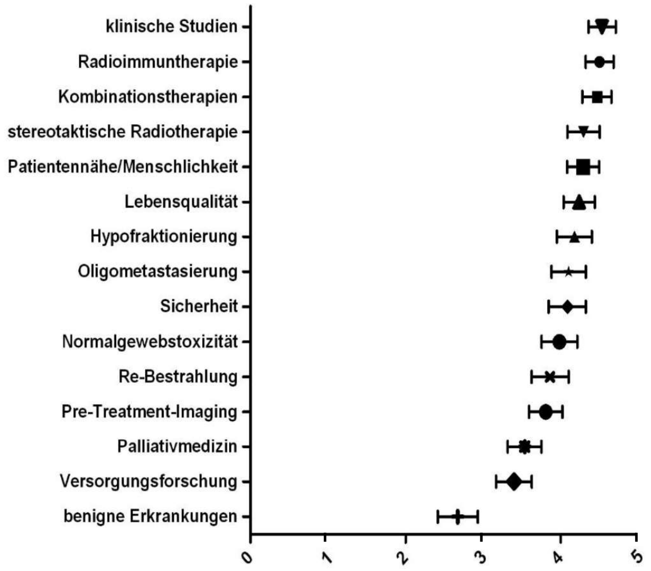

Medizin/Patientenversorgung

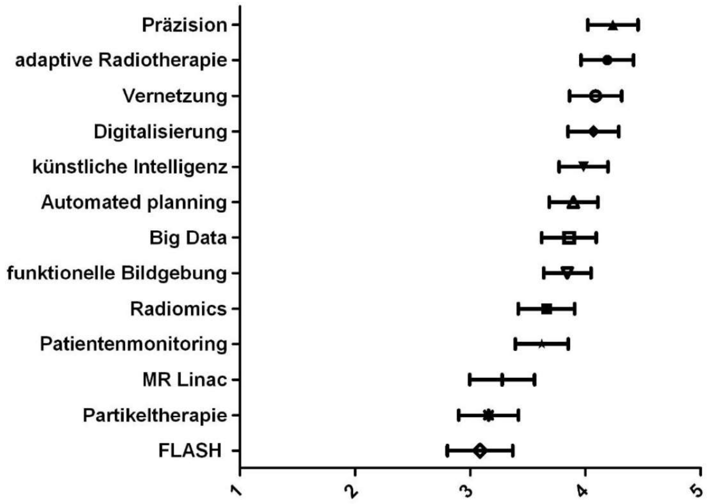

Physik/Technik

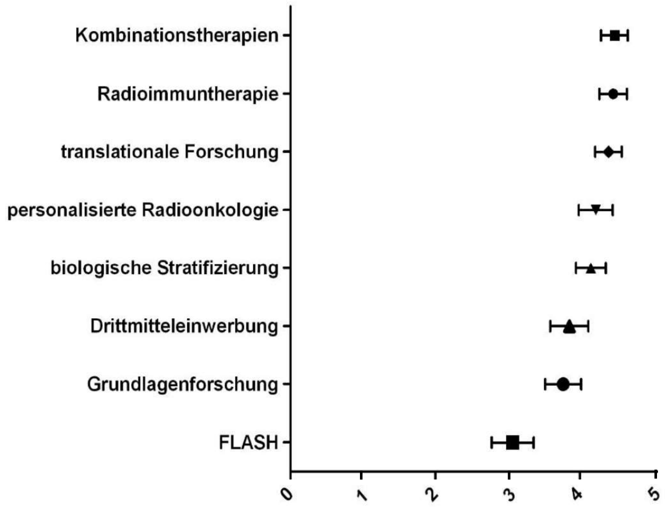

Strahlenbiologie

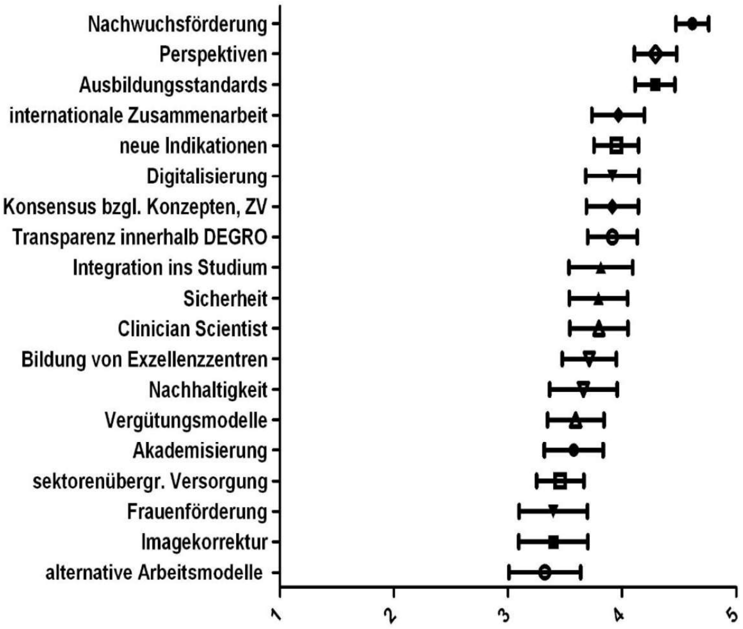

Fachgesellschaft

Zur Methodik:

1. Zusendung von 5 Schlüsselwörtern:
   29 Rückmeldungen von 81 Teilnehmern
2. Gruppierung der Schlüsselwörter
3. Online-Gewichtung via Survey-Monkey auf einer Skala von 1 (unwichtig) bis 5 (sehr wichtig):
   63 Teilnehmer davon 40 Medizin, 12 Biologie, 10 Physik, 11 AKRO-Mitglieder

Ablauf der Gruppenarbeit

Zeit	Ablauf	Hinweise
12:45 – 13:00	Moderatorenimpulse II im Plenum (nur Moderatoren)	
13:00 – 13:30	Ankommen, Begrüßung, Poster betrachten. Ziel der nächsten 2 Stunden.	
	2 – 3 weitere Schlüsselwörter definieren.	TN schreiben max. 2 Karten. Auf Flip kleben. Auswahl: Jeder bekommt 3 Punkte.
	Diskussion der Schlüsselwörter und ggf. der sonstigen Vorabinfos anhand Leitfragen.	Konkretisierung. Focus? Zusammenhänge? Kausalitäten?
13:30 – 14:15	2 Visionen erarbeiten.	2 Kleingruppen: 1 x 5 TN, 1 x 4 TN. Pro Kleingruppe 1 Vision.
	Präsentation der Visionen gegenseitig. Feedback.	
	„Nachschärfen" der Visionen in den Kleingruppen anhand Feedbacks.	
	Auswahl einer Vision.	Über Punktabfrage (jeder TN einen Punkt) oder Diskussion.
14:15 – 14:45	Diese eine Vision nach den Teilbereichen Patientenversorgung, Forschung, Weiterbildung und Fachgesellschaft interpretieren.	Pro Teilbereich eine Kleingruppe: Drei 2er Teams, ein 3er Team.
	Präsentator für die gewählte Vision bestimmen.	

© being to inspire

Ergebnissicherung und Konsensfindung im Plenum

Zeit	Ablauf	Hinweis
14:45 – 15:30	Bei Kaffee und Kuchen Erklärung der Methode World-Café im Plenum.	
	Gruppenpräsentationen 1 – 9 im World-Café.	Je Präsentation 3 Min.
	„Nachschärfen“ aufgrund Feedbacks in World-Café in den Gruppen (→ Gruppenräume).	
15:30 – 16:30	9 Visionen + Konkretisierung werden im Plenum an Moderationswände gepinnt.	Pro Vision eine Moderationswand.
	Aus 9 Visionen 3 machen. Moderation durch ausgewählte Moderatoren.	Drei Gruppen à 30 TN. Gr. A (Gr. 1-3): Vision 1-3, Gr. B (Gr. 4-6): Vision 4-6, Gr. C (Gr. 7-9): Vision: 7-9.
	Jede Gruppe präsentiert Ihre Vision im Plenum. Feedback.	
16:30 – 16:40	Die 3 Visionen sind das Ergebnis, mit dem weitergearbeitet wird, oder..	Ausrichtung der 3 Visionen wird wahrscheinlich ähnlich sein.
	Auswahl einer oder mehrerer finaler Visionen. Commitment mit Gruppenfoto.	Über Punktabfrage (jeder TN einen Punkt) oder Diskussion.
16:40 – 17:00	Wie geht es weiter?	
	Schlussworte	

© being to inspire

Visionen der Kleingruppen			Kondensate
Evidenz Schaffen<br><br>90% der Patienten sind in Studien! (prospektiv, Register, Material, Uniklinik & Praxis)<br><br>Parameter in Physik heranziehen + standardisieren<br><br>Jeder bestrahlter Patient wird prospektiv standardmäßig erfasst: klinisch, biologisch, physikalisch<br><br>„Cloud" Daten anonymisiert in Berlin<br>Zentrale Einwilligung für die DEGRO-Datenbank (Strahlenschutz)	Smarte Radioonkologie durch optimale<br><br>Vernetzung	Gemeinsam. Fokussiert, nachhaltig, strukturiert für uns und unsere Patienten<br><br>Fokussierung<br>• Schwerpunkte / (Exzellenz-)zentren<br>• Forschung<br>• Added Value (Technik)<br>• Kommunikation<br>• Effizienz<br>• Kräfte Bündeln<br><br>Gemeinsam:<br>• Reputation des Fachs<br>• Patientenversorgung<br>• Sichtbarkeit<br><br>Nachhaltigkeit ⟷ Struktur<br><br>Nachhaltigkeit<br>• Teamarbeit<br>• Ausbildung<br>• Mentoring<br>• Förderprogramme<br>• Selbstbewusstsein<br>• Verantwortung für Pat. übernehmen<br><br>Struktur<br>• Ausbildung<br>• SOP / Leitbild für Mentoring & Ausbildung<br>• Transparenz<br>• Vernetzung	Gemeinsam fokussiert Evidenz schaffen
Im Team gegen Krebs<br><br>• Menschlich<br>• Wissenschaftlich<br>• Innovativ	Innovativ<br>Interdisziplinär<br>Personalisiert<br><br>Für unsere Patienten<br><br>Personalisierte Radioonkologie ⟷ Interdisziplinarität<br><br>Patient<br><br>Innovation	Arbeitsplätze in Zukunft<br><br>• Fordern & Fördern: Freistellung/Weiterbildung/Forschung, Zielvereinbarungen<br>• Familienfreundlichkeit: Flexible Arbeitsmodelle, z. B. durch Digitalisierung<br>• Entlastung bei eigentlich „nicht-ärztlichen" Tätigkeiten<br>• Aufwertung/Akademisierung d. TA's<br>• Konkurrenzfähigkeit d. ausbildenden vs. nicht-ausbildenden Institution „Ablöse"<br>• Know-How-Sicherung	Innovative Radioonkologie im Team<br>• Präzise<br>• Personalisiert<br>• Menschlich
Mit hochqualifiziertem Nachwuchs innovative Therapien mit und für unsere Patienten entwickeln	Radioonkologie ist ein Fach mit „Strahlkraft"<br><br>Unsere Vision: diese Strahlkraft von Innen heraus stärken:<br><br>Durch:<br>• Forschung, Personalisierte Medizin<br>• Fachgesellschaft<br>• Weiterbildung/Ausbildung<br>• Neue Laufbahnmodelle<br><br>Nach Außen:<br>• Stakeholder (Finanzförderung, politische/regulat. Instanzen)<br>• Andere Fächer (med. + nicht-med.)<br><br>Fachübergreifender Player/Akteur vertreten<br>→ Interaktionsplattformen schaffen<br><br>=> Übersetzung in einen substantiellen Patientennutzen	Begeisternd gemeinsam Zukunft gestalten!<br>Die Radioonkologie nimmt eine zentrale Rolle in der interdisziplinären Onkologie ein und gestaltet für alle Berufsgruppen eine strukturierte und zukunftsorientierte Perspektive in Patientenbehandlung, Ausbildung, Karriere und Forschung.	Begeisternd gemeinsam die Zukunft der Radioonkologie gestalten!<br><br>Mit hochqualifiziertem Nachwuchs innovative Therapien mit und für unsere Patienten entwickeln

Visionsentwicklung der Strahlentherapie & Radioonkologie in Deutschland. In 9 Kleingruppen à 8 Vertreter der AKRO und jDEGRO aus Medizin, Biologie und Physik wurden die Visionen der Kleingruppen zu drei Visionen fusioniert und final abgestimmt auf „Innovative Radioonkologie im Team – Präzise, Personalisiert, Menschlich".
