# Supplementary material for: Innovative radiation oncology Together – Precise, Personalized, Human: Vision 2030 for radiotherapy & radiation oncology in Germany
Source: Strahlenther Onkol. 2021 Sep 13;197(12):1043–8. doi: 10.1007/s00066-021-01843-9 (PMC8604860; doi:10.1007/s00066-021-01843-9)
Supplement: Supplementary file 2 — Supplement 2: Supporting statements from ARO, BVDST, DeGBS and DGMP. [file 66_2021_1843_MOESM2_ESM.pdf]

BVDST Berufsverband Deutscher Strahlentherapeuten e.V.

BVDST · Geschäftsstelle · Carmerstraße 2 · 10623 Berlin

Vorstand
Vorsitzender
Prof. Dr. F.-J. Prott

Berufsverband Deutscher
Strahlentherapeuten e.V.
Geschäftsstelle
Carmerstraße 2
10623 Berlin

Tel.: (030) 31 00 77 14-0
Fax: (030) 31 00 77 14-77
info@bvdst.de
www.bvdst.de

Geschäftszeiten
Dienstag – Donnerstag
09:00 – 15:30 Uhr

Vorstand des BVDST
Vorsitzender
Prof. Dr. F.-J. Prott,
Wiesbaden

Stellv. Vorsitzender
Dr. W. Leßmann,
Leverkusen

Schatzmeister
Priv. Doz. Dr. M. van Kampen,
Frankfurt/ Main

Schriftführerin
Priv. Doz. Dr. A. Fahrig,
Bamberg

1. Beisitzer
Prof. Dr. O. Micke,
Bielefeld

2. Beisitzer
Univ. Prof. Dr. R. Engenhart-Cabillic,
Marburg-Gießen

3. Beisitzer
Prof. Dr. P. Feyer,
Berlin

4. Beisitzer
Prof. Dr. St. Höcht,
Saarlouis

Ihr Zeichen/Mitgliedsnummer	Unser Zeichen	Datum
		27.04.2021

Zukunftsvision deutsche Strahlentherapie

Sehr geehrte Alumni der Jungen DEGRO,

der Vorstand des Berufsverbandes Deutscher Strahlentherapeuten BVDST begrüßt und unterstützt die Bestrebungen der Jungen DEGRO eine gemeinsame Zukunftsvision der deutschen Strahlentherapie zu entwickeln. Diese Anstrengungen können nachhaltig das Fach und die Zusammenarbeit zwischen Praxen, Krankenhausabteilungen und Universitätskliniken stärken und darüber hinaus wird es möglich sein, Menschlichkeit und Wünsche/Bedürfnisse der Patienten im Fokus zu behalten.
Es gelingt Ihnen in Ihrem Manuskript eine gute Definition für die Schlagwörter aus der Zukunftsvision zu geben und mit Beispielen zu unterfüttern, eine Programmatik darzulegen und damit das erklärte Ziel dieses Manuskriptes zu erreichen.
Gerne möchten wir bei dieser Gelegenheit den Fokus auf einen Aspekt legen, der uns in diesem Zusammenhang aus der Sicht des BVDST besonders wichtig ist: die weitere Sicherung der Wirtschaftlichkeit der Strahlentherapie auf der Basis von Versorgungsforschung.
Wir halten die Thematik für außerordentlich wichtig, da auf der einen Seite immer mehr Tumortherapien in hypofraktionierten Behandlungsschemata durchgeführt werden, auf der anderen Seite aber nach wie vor die Abrechnung, zumindest bei den gesetzlich versicherten Patienten, pro Fraktion erfolgt.
Mit dieser Thematik, denke ich, können wir uns hervorragend einbringen. Hier kann der Berufsverband einen wertvollen Beitrag leisten und möchte dieses auch gerne tun.

mit den besten Wünschen für ein gutes Gelingen

Prof. Dr. Prott
Vorsitzender des BVDST

Arbeitsgemeinschaft Radiologische Onkologie

ARO • Reinhardtstraße 47 • 10117 Berlin

DEGRO working group „Young DEGRO“
attn. Prof. Dr. med. Maximilian Niyazi

via email to:
maximilian.niyazi@med.uni-muenchen.de

Prof. Dr. med. Mechthild Krause
Speaker oft he ARO working group
+49 (351) 458 21 43
mechthild.krause@uniklinikum-dresden.de

ARO-Office
Reinhardtstraße 47 | 10117 Berlin
+49 (30) 84 31 89 89
+49 (30) 84 31 89 89
office@aroonline.de
www.aroonline.de

Berlin | 29. April 2021

Letter of Support: Manuscript “Innovative Radiation Oncology Together – Precise, Personalised, Human. Vision 2030 for Radiotherapy & Radiation Oncology in Germany” (AKRO/yDEGRO)

The Working Group Radiation Oncology (ARO) of the German Cancer Society is fully supporting the vision "Innovative Radiation Oncology Together – Precise, Personalised, Human” developed by the AKRO and young DEGRO representatives.

As highlighted in the manuscript the term "Innovative Radiation Oncology" represents the design and initiating of clinical trials and translational research. This is in line with the main goal of the ARO working group, which is responsible for the support and organization of clinical studies in radiation oncology within the German Cancer Society.

Thus, the ARO group supports and endorses the initiative and the vision for radiotherapy & radiation oncology in Germany.

Prof. Dr. med. Mechthild Krause
Speaker of the ARO working group

Board:
Prof. Dr. M. Krause, Dresden (Sprecherin); Prof. Dr. U. Nestle, Mönchengladbach (stellvertretende Sprecherin);
PD Dr. D. Böhmer, Berlin; Prof. Dr. E. Fokas, Frankfurt/Main; Prof. Dr. S. Höcht, Saarlouis;
Prof. Dr. S. Marnitz-Schulze, Köln; Prof. Dr. H. Schmidberger, Mainz

Deutsche Gesellschaft für Medizinische Physik e.V.

BOARD OF DIRECTORS

President:
Prof. Dr. Dimos Baltas

Vice President:
Prof. Dr. M. Fiebich

Vice President:
Prof. Dr. Mark E. Ladd

Treasurer:
Dr. Jens Heufelder

Secretary:
Prof. Dr. Christoph Bert

Chair of Departments:
Dr. Daniela Schmitt
Dr. Josefin Ammon
Prof. Dr. Dr. U. Hoppe

Public Relations Officer:
Prof. Dr. M. Buchgeister

DGMP | Office | Ernst-Reuter-Platz 10 | 10587 Berlin | Germany

yDEGRO alumni representatives
Prof. Dr. Maximilian Niyazi and Dr. David Krug

31. Mai 2021

CONTACT

DGMP Office

Dr. Erik Gührs
phone: +49 (0) 30 398 351 90
fax: +49 (0) 30 916 070 22
e-mail: guehrs@dgmp.de

Julia Lepinat
phone: +49 (0) 30 916 070 39
fax: +49 (0) 30 916 070 22
e-mail: office@dgmp.de

Vision 2030 for Radiotherapy & Radiation Oncology in Germany

Dear Sir or Madam,

The German Society of Medical Physics (DGMP) emphasises its support and partnership for the implementation of the DEGRO "Vision 2030 for Radiotherapy & Radiation Oncology in Germany". For us, all programme points dealing with standardisation and systematic data collection and analysis are of high importance for the whole field of radiotherapy in research and clinical application integrated in the international setting. The points "Development of national quality standards on implementing new technologies" and "Standardised procedures in data acquisition of clinical data and study registries" may serve as examples for the aforementioned. Main topics for our joint effort are also related to the development of "Standardised and evidence-based technological quality requirements".

Several aspects like "Promotion of industrial technology development based on clinical needs and evidence" and "Establishment of national concepts and collaborations for machine learning and artificial intelligence" clearly need additional collaborations with the industry partners in medical technology and with computer scientists.

The second main item deserving our corporate activities is related to the "Together" part of the vision, which is essential for long term success and satisfaction in all radiation oncology professions, i.e., radiation oncologists, medical physicists, radiation biologists, radiation therapists and nursing staff. Additionally, scientific and clinical research and exchange programmes for all professions are clearly needed for personal and scientific development and the integration in an international field of expertise as well as "Expanding professorships of basic research and of translational research".

With kind regards,

Prof. Dr. Dimos Baltas
President of DGMP

Dr. Daniela Schmitt
Department Chair Therapeutic Methods

Deutsche Gesellschaft für Biologische Strahlenforschung e.V.

An

Dr. David Krug

PD Dr. Maximilian Niyazi

Junge DEGRO

1. Vorsitzende:
Prof. Dr. Verena Jendrossek
Institut für Zellbiologie (Tumorforschung)
Universitätsmedizin Essen,
Universitätsklinikum und Medizinische Fakultät der Universität Duisburg-Essen
Virchowstrasse 173

45147 Essen

Tel.: 0201-7233380

06. April 2021

Betreff: Stellungnahme des DeGBS-Vorstands zum Positionspapier der jungen DEGRO zur Zukunftsvision Innovative Radioonkologie 2030

Sehr geehrter Herr Dr. Krug,

der Vorstand der DeGBS begrüßt und unterstützt die Bestrebungen der jungen DEGRO zur Etablierung von innovativen individualisierten Therapien in der Präzisions-Strahlentherapie 2030 durch die Zusammenarbeit in interdisziplinären Teams (u.a. mit Einbeziehung von Naturwissenschaftlern aus der Biologie und Physik, aber auch nicht-akademischen Disziplinen), translationale Forschungsansätze sowie die Entwicklung innovativer molekularer Optionen und von Biomarkern, die darüber hinaus Menschlichkeit und die Wünsche/Bedürfnisse der Patienten im Fokus behalten.

Es gelingt Ihnen im Manuskript, eine gute Definition für die Schlagwörter aus der Zukunftsvision zu geben, und mit Beispielen zu unterfüttern und damit das erklärte Ziel dieses Manuskriptes zu erreichen.

Gerne möchten wir bei dieser Gelegenheit den Fokus auf einen Aspekt legen, der uns in diesem Zusammenhang aus Sicht der DeGBS besonders wichtig ist: wir sind der Ansicht, dass die Umsetzung der Vision tatsächlich eine Team-Anstrengung erfordert, die nicht nur ein intensives multidisziplinäres Training und Mentoring von *Clinician Scientists*, sondern auch von *Medical Scientists*, *Medical Physicists*, und *Medical Data Scientists* in den vielfältigen grundlagenwissenschaftlichen, translationalen und klinischen Aspekten der Präzisionsmedizin und Radioonkologie sowie darüber hinaus auch die Förderung der Interaktion und Zusammenarbeit zwischen den Disziplinen einbeziehen sollte. Diese Anstrengungen wird der Vorstand der DeGBS gerne unterstützen.

Viel Erfolg und herzliche Grüße,

(Prof. Dr. Verena Jendrossek, 1. Vorsitzende)
