# Supplementary material for: Innovative radiation oncology Together – Precise, Personalized, Human: Vision 2030 for radiotherapy & radiation oncology in Germany
Source: Strahlenther Onkol. 2021 Sep 13;197(12):1043–8. doi: 10.1007/s00066-021-01843-9 (PMC8604860; doi:10.1007/s00066-021-01843-9)
Supplement: Supplementary file 3 — Supplement 3: Development process of German radiotherapy & radiation oncology vision including embedded hyperlinks. [file 66_2021_1843_MOESM3_ESM.pdf]

Initiation & Preparation

11.2018 – Drafting of yDEGRO[1] white paper on promotion of young clinicians and scientists in radiation oncology

02.2019 – Consolidation of white paper by AKRO[2] and initiation of vision development through a yDEGRO/AKRO retreat

06.2019 – Agreement on objectives/process of vision development and target audience of a yDEGRO/AKRO retreat with DEGRO[3] board

08.2019 – Instruction of retreat participants; provision of background material incl. visions of related organisations; and online survey among participants on vision keywords & weighting

yDEGRO/AKRO Retreat, November 15th 2019, Frankfurt

- Keynotes by DEGRO, DKTK[4] and ESTRO[5] on experience and lessons learned during vision development
- Vision development in 9 subgroups, each consisting of 8 AKRO and yDEGRO representatives from medicine, biology and physics
- Vision presentations applying the World-Café-Method followed by subgroup revisions
- Consolidation of 9 visions into 3 visions by 3 groups
- Plenum discussions on 3 consolidated visions and voting on the final vision
- Reporting of yDEGRO/AKRO retreat and drafting of vision interpretation & programme by yDEGRO alumni respresentatives[6]

Vision Interpretation & Programme

08.2020 – Drafting of interpretation & programme in 7 groups each consisting of 5 yDEGRO alumni representatives

11.2020 – Finalisation of draft interpretation & programme by yDEGRO/AKRO retreat participants

12.2020 – Agreement on final interpret. & programme with DEGRO board; commentary of associated boards of BVDST[7], DGMP[8], DeGBS[9], ARO[10]

05.2021 – Dissemination of vision outcome; preparation of programme implementation by yDEGRO alumni

Fig. 1 Development process of German radiotherapy & radiation oncology vision "Innovative Radiation Oncology Together – Precise, Personalized, Human".
[1]German Society for Radiation Oncology (*DEGRO*) working group of young clinicians and scientists and its [6]Alumni representatives as a link between young scientists and executives/leaders in radiotherapy & radiation oncology in Germany. [2]Representation of associate and full professors of DEGRO. [3]German Society for Radiation Oncology. [4]German Consortium for Translational Cancer Research. [5]European Society for Radiotherapy & Oncology. [7]Professional Association of German Radiation Oncologists. [8]German Society of Medical Physics. [9]German Society of Radiobiology Research. [10]Working Group Radiation Oncology of German Cancer Society.
